# Suppression of Rac1 Signaling by Influenza A Virus NS1 Facilitates Viral Replication

**DOI:** 10.1038/srep35041

**Published:** 2016-11-21

**Authors:** Wei Jiang, Chunjie Sheng, Xiuling Gu, Dong Liu, Chen Yao, Shijuan Gao, Shuai Chen, Yinghui Huang, Wenlin Huang, Min Fang

**Affiliations:** 1 CAS Key Laboratory of Pathogenic Microbiology and Immunology, Institute of Microbiology, Chinese Academy of Sciences, Beijing 100101, China; 2 University of Chinese Academy of Sciences, Beijing, China; 3 State Key Laboratory of Oncology in South China, Cancer Center, Sun Yat-sen University, Guangzhou 510060, China; 4 College of Life Science and Bioengineering, Beijing University of Technology, Beijing 100124, China; 5 Key Laboratory of Tumor Targeted Drug in Guangdong Province, Guangzhou Double Bioproducts Co., Ltd., Guangzhou, China; 6 International College, University of Chinese Academy of Sciences, Beijing, China

## Abstract

Influenza A virus (IAV) is a major human pathogen with the potential to become pandemic. IAV contains only eight RNA segments; thus, the virus must fully exploit the host cellular machinery to facilitate its own replication. In an effort to comprehensively characterize the host machinery taken over by IAV in mammalian cells, we generated stable A549 cell lines with over-expression of the viral non-structural protein (NS1) to investigate the potential host factors that might be modulated by the NS1 protein. We found that the viral NS1 protein directly interacted with cellular Rac1 and facilitated viral replication. Further research revealed that NS1 down-regulated Rac1 activity via post-translational modifications. Therefore, our results demonstrated that IAV blocked Rac1-mediated host cell signal transduction through the NS1 protein to facilitate its own replication. Our findings provide a novel insight into the mechanism of IAV replication and indicate new avenues for the development of potential therapeutic targets.

Influenza A virus (IAV) is an enveloped, segmented, negative-strand RNA virus in the *Orthomyxoviridae* family that was responsible for the devastating Spanish flu and the 2009 pandemic^1^,^2^. The ability of IAV to exchange RNA segments among currently circulating human and animal virus serotypes stresses that IAV remains a world-wide threat^3^,^4^,^5^. Moreover, increasing evidence indicates that IAV can exploit host factors to enhance its infectivity and propagation, and more than 476 cellular factors are involved in this network^6^,^7^,^8^. Knowledge of the cellular factors that facilitate virus replication will enhance our understanding of IAV-mediated pathogenesis and provide potential antiviral targets to spur the development of innovative treatments to prevent various types of IAV cross-species transmission.

Non-structural protein 1 (NS1) is a key multifunctional virulence factor of influenza A viruses that plays distinct role in viral replication and disease progression^9^,^10^. NS1 is composed of an RNA binding domain for interactions with RNA and an effector domain to mediate interactions with cellular proteins^11^,^12^. The key functions of NS1 include the regulation of viral protein synthesis via mRNA splicing and translation^13^, interference with host restriction factors^14^,^15^,^16^, inhibition of the antiviral type 1 interferon response^17^,^18^,^19^, and suppression of NLRP3 inflammasome-mediated IL-1β secretion^20^,^21^. Like PB1-F2^22^ and PA-X^23^,^24^, NS1 protein is not included in the viral particle, which suggests that it might be special compared to other virion proteins.

Rac1 is a small GTPase that is primarily localized in the cytoplasm, although nuclear Rac1 has been reported^25^,^26^,^27^. It is a multifunctional protein involved in numerous cellular processes that are critical for cell ruffling, adherence junction formation, cell motility, polarity and proliferation^28^. Similar to most GTPases, Rac1 functions as a molecular switch between GTP and GDP and is regulated by numerous guanine nucleotide exchange factors (GEFs) and several GTPase-activating proteins (GAPs)^29^,^30^,^31^. Several studies have suggested that the subcellular localization of Rac1 plays a major role in the regulation of virus entry, replication and release^32^,^33^,^34^ and that the inhibition of Rac1 leads to enhanced virus production^35^.

Protein ubiquitination and SUMOylation are vital post-translational modifications in numerous signaling pathways^36^,^37^,^38^,^39^. The ubiquitination reaction consists of the covalent attachment of ubiquitin (an 8-kDa polypeptide) to lysine residues in target proteins^40^,^41^. Additionally, small ubiquitin-like modifier (SUMO) proteins 1, 2 and 3 can be covalently conjugated to specific lysine residues in target proteins by a process termed SUMOylation^42^,^43^. This conserved post-translational modification was initially reported in 1996 and has since emerged as an important regulatory mechanism in cell physiology, particularly in nuclear signaling, transport, transcription and DNA replication/repair^44^,^45^. Recently, more detailed studies have indicated that most members of the small GTPase family, including Rac1, can be modulated by these post-translational modifications, which differ from the modification mediated by GEFs, GAPs, and RhoGDI, to controll cycling between the active and inactive states^25^,^46^,^47^,^48^,^49^.

Recent studies have described novel cellular factors and protein-protein interactions that are important for IAV replication; however, many fundamental processes in the complete viral replication cycle remain uncharacterized^50^,^51^,^52^. Ehrhardt *et al*. showed that Rac1 inhibition led to enhanced virus production^35^. In this study, we found that the viral NS1 protein directly interacted with cellular Rac1 and facilitated viral replication. We showed that NS1 down-regulated Rac1 activity via post-translational modification. To our knowledge, this study is the first report to demonstrate that the IAV NS1 protein can directly interact with the cellular protein Rac1 and modulate its activity via post-translational modifications to regulate IAV replication. Thus, our results provide a new perspective for understanding influenza virus-host interactions.

## Materials and Methods

### Cell culture and transient transfection

A549 cells (human airway epithelial cells) were obtained from the American Type Culture Collection (ATCC) and cultured in RPMI 1640 medium. A human embryonic kidney cell line (293T) and Madin-Darby canine kidney cells (MDCK) were obtained from the ATCC and cultured in Dulbecco’s modified Eagle’s medium (DMEM). The RPMI 1640 and DMEM media were supplemented with 10% heat-inactivated fetal bovine serum (Gibco, Paisley, UK). Plasmid transfections were performed using the Effectene Transfection Reagent (Qiagen, Germany) according to the manufacturer’s instructions.

### Plasmids and antibodies

H1N1 NS1 cDNA was derived from strain A/WSN/33 and sub-cloned into the eukaryotic expression vector pcDNA3.0-flag. Using published sequences, we obtained full-length human Rac1 cDNA by reverse-transcription PCR; then, full-length Rac1 was cloned into the pcDNA4.0-myc and the pEGFP-N1 vectors. The Rac1 mutations K147R, K153R, K166R, K183R, K184R, K186R, K188R, ΔSUMO and ΔWT were all cloned into the pcDNA4.0-myc vector. Ubiquitin (Ub)-conjugated HA (HA-Ub), HA-K48-Ub, and HA-K63-Ub were kindly provided by Xin Ye (Institute of Microbiology, Chinese Academy of Sciences, Beijing, China). HA–SUMO-1 was cloned into the pXJ40-HA vector. The Ubc9 plasmid was kindly provided by Hong Tang (Institute of Biophysics, Chinese Academy of Sciences, Beijing, China).

The mouse anti-flag (M2; F3165) antibody was purchased from Sigma (MO, USA). The mouse anti-HA (sc-7392), anti-Myc (sc-40), anti-influenza A NP (sc-101352), anti-influenza A NS1 (sc-130568) antibodies, anti-β-actin (sc-130656) antibodies and the rabbit anti-Rac1 (sc-95) were obtained from Santa Cruz Biotechnology (CA, USA). The goat anti-HA-tag polyclonal antibody was purchased from GenScript Corporation (NJ, USA). The TRITC-conjugated anti-mouse IgG was purchased from Zhongshan Golden Bridge Biotechnology (Beijing, China).

### Immunofluorescence assay

To determine the positive rate of NS1 protein expression in the over-expression stable cell lines, 10^6^ cells were collected and fixed in 4% paraformaldehyde for 15 minutes at room temperature. Then, the cells were permeabilized with 0.2% Triton X-100 for 5 minutes. After blocking in 5% BSA for 30 minutes, the cells were incubated for 35–40 minutes with primary anti-flag antibodies at 37 °C. After washing with PBS, the cells were incubated for 40 minutes with TRITC conjugated secondary antibodies at 37 °C. Then, fluorescence-activated cell sorting (FACS) was employed to select cells with red fluorescence. The proportion of NS1-positive cells was calculated as the percentage of M2 within the gated cell population (M1). At least 20,000 cells were counted per sample. The data analysis was performed using ModFit LT version 2.0 to determine the infection efficiency.

### Wound healing migration assay

Transfected cells grown in complete medium were seeded into six-well plates. After the cells grew to confluence, wounds were made using 10 μl sterile pipette tips. Then the cells were washed with PBS and fresh medium was added. After incubation for 24 hours at 37 °C, the cells were fixed and photographed.

### Chamber cell-migration assay

Cell migration was assayed in Boyden chambers [8.0-μm-pore-size polyethylene terephthalate membrane with a Falcon cell-culture insert (Becton Dickinson)]. The cells were trypsinized and counted. Cell suspensions (5 × 10^4^ to 10 × 10^4^ cells in 300 μl of serum-free medium) were added to the upper chamber, and 500 μl of the appropriate medium was added to the lower chamber. The transwells were incubated for 24 hours at 37 °C. The cells on the inside of the transwell inserts were removed with a cotton swab, and the cells on the underside of the insert were fixed and stained. Photographs were taken of three random fields, and the cells were counted to calculate the average number of transmigrated cells.

### Rac1 activation and the PAK-PBD pull-down assay

Rac1 activation was determined with the Rac1 pull-down activation assay. Transfected A549 cells were grown to confluence in 100 mm dishes and then cultured in serum-free medium for 24 hours. The cells were stimulated with 10% FBS for 15 minutes and then washed with ice-cold PBS. The GTP-Rac1-enriched lysates were incubated with 20 μl of the PAK-PBD reagent (a GST fusion protein); this fusion allows the “pull-down” of the PAK-PBD/GTP-Rac1 complex with glutathione affinity beads because the beads bind to Rac1 only in its GTP-bound form (GST-PAK-PBD). GST alone was used as a control. This assay provides a method to quantify Rac1 activation in the cells. The amount of activated Rac1 was determined by the western blotting of the precipitated material using a Rac1-specific antibody.

### Virus generation and infection experiment

The recombinant influenza virus A/WSN/33 (H1N1) was generated using a 12-plasmid IAV reverse-genetics system and propagated in MDCK cells. An influenza virus A/WSN/33 strain that lacked the NS1 gene (NS1^−^ H1N1 virus) was generated using pPolI-NS2 to alter the pPolI-NS reverse genetics^6^. The viral titer was determined by plaque assay or by indirect immunofluorescence microscopy with an anti-NP antibody in MDCK cells^15^,^53^.

For virus infection, the cells were mock-infected or infected with virus at different multiplicities of infection (MOI). After 1 hour of incubation to allow virus adsorption, the cells were cultured in complete medium and harvested at various time points post-infection

### GST pull-down and co-immunoprecipitation (IP) analysis

GST and the GST fusion protein were purified from *E. coli* BL21 cells (DE3) using glutathione Sepharose 4B beads (Amersham Biosciences, Uppsala, Sweden). An equal amount of GST or the GST fusion protein bound to glutathione beads was incubated with the lysates from transiently transfected 293T cells in NP-40 lysis buffer for 4 hours at 4 °C. Then the beads were washed three times with PBS containing 0.1% Triton X-100. The bound proteins were eluted by boiling in 2 × SDS loading buffer and analyzed by western blotting.

For the co-immunoprecipitation experiments, total cell lysates from transfected 293T cells in lysis buffer (1% Triton X-100, 150 mM NaCl, 20 mM HEPES pH 7.5, 10% glycerol, 1 mM EDTA, and protease inhibitors) were incubated with antibody at 4 °C for 2 hours. Protein G agarose beads (Sigma, USA) were added, and the samples were incubated at 4 °C overnight. The beads were washed three times with lysis buffer and boiled in 2 × SDS loading buffer for 5 minutes. The samples were analyzed by western blotting.

### Subcellular localization and immunofluorescence assay

To determine the co-localization of the NS1 and Rac1 proteins, A549 cells were co-transfected with the pEGFP-NS1 and pcDNA4.0-myc-Rac1 plasmids. After 24 hours, the cells were washed three times with PBS, fixed in 4% paraformaldehyde for 15 minutes at room temperature, and permeabilized with 0.2% Triton X-100 for 5 minutes. After blocking with 5% BSA for 30 minutes, the cells were incubated for 1 hour with an anti-myc monoclonal antibody at room temperature. After washing with PBS, the cells were incubated for 1 hour with a TRITC-conjugated anti-mouse IgG antibody and then visualized with an Olympus confocal microscope.

### miRNA expression vectors

Expression vectors that encoded shRNAs specific for Rac1 or a control were constructed using psiSTRIKE^TM^ vectors (Promega) according to the manufacturer’s protocol. Primer sequences for the control shRNA and shRac1-specific shRNAs were designed according to the instructions provided by the manufacturer and are shown in Table 1. The primers were annealed to form double-stranded DNA and then inserted into the psiSTRIKE^TM^ vectors. Bacteria were transformed with the resulting constructs, and a reasonable number of colonies were obtained. The resulting shRNA-encoding expression vectors were confirmed by digestion with the restriction enzyme *PstI* according to the manufacturer’s protocol. *C. elegans* miR239b was used as a negative control.

### Plaque assay

Ten-fold serial dilutions of the different virus types were used to infect confluent MDCK cells in 24-well plates for 1 h at 37 °C. The virus inocula were removed by washing with PBS. The cell monolayers were overlaid with an agar medium (DMEM supplemented with 1% low melting point agarose and 1 μg/ml of TPCK-treated trypsin) and incubated at 37 °C for 3 days. Then, the plates were fixed with 4% paraformaldehyde for 1 h, and the agarose was carefully removed. Staining buffer (0.1% crystal violet and 20% ethanol in water) was added for 10 min, followed by washing before the virus titer was determined. All data were expressed as the mean of three independent experiments.

### Statistical analysis

One representative western blot is shown for three similar independent experiments. The protein expression levels were quantified using the Image J software, and the results were presented as the mean ± SD of triplicate experiments. The data were evaluated using a *t*-test with the SPSS 11.5 software (SPSS, Inc., Chicago, IL, USA). P < 0.05 was considered statistically significant.

## Results

### NS1 alters cell morphology and inhibits cell migration

**Figure 1:**
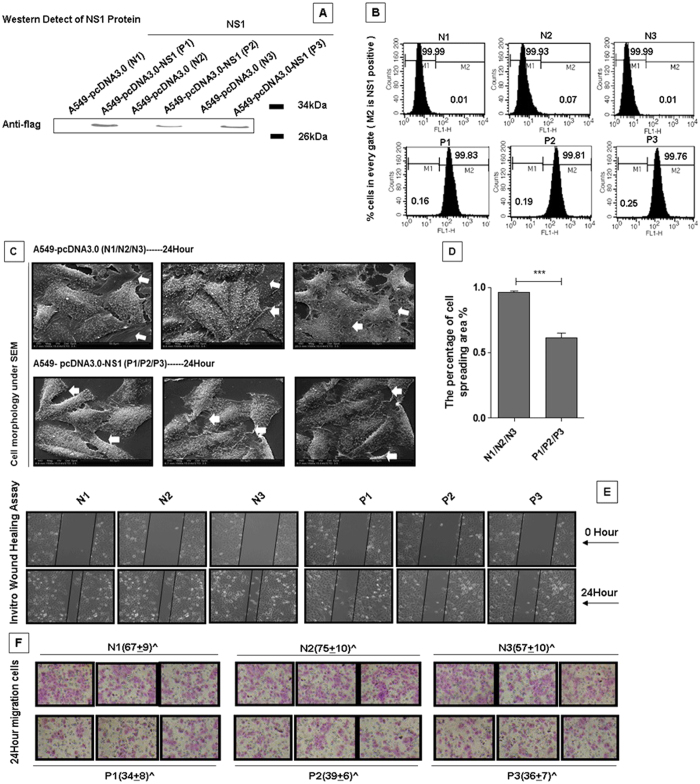
NS1 modulates cell morphology and migration. (**A**) Generation of the NS1 stable cell lines. A549 cells were transfected with the pcDNA3.0-flag or pcDNA3.0-flag-NS1 plasmid. After selection in 800 mg/L G418 for 3 weeks, the NS1 expression levels were detected in the stably transfected cell lines by western blotting using anti-flag antibody. (**B**) The NS1 expression levels in the NS1-positive cell lines were determined by fluorescence-activated cell sorting (FACS). The indicated cell lines were fixed, permeabilized, and stained for Flag-NS1. ModFit LT version 2.0 was utilized to determine the percentage of NS1-positive cells. (**C**) Scanning electron microscopy (SEM) showed the morphological differences between the control cell lines (N1, N2, and N3) and the NS1 over-expressing cell lines (P1, P2, and P3). The cell lines were cultured for 24 hours, and then fixed, dried, sprayed with metal and photographed by SEM. White arrows indicate the cellular lamellipodia. (**D**) The percentage of the cell spreading area. Image J software was used to analyze the cell spreading area. The results are presented as the mean ± SD of triplicate experiments (***p < 0.001 relative to the control group). The data were evaluated using a *t*-test with the SPSS 11.5 software program (SPSS, Inc., Chicago, IL, USA). (**E**) A wound healing assay was performed to detect the migration ability in the two different cell lines. The indicated cell lines were seeded into six-well plates. After the cells grew to confluence, wounds were made using 10 μl sterile pipette tips. Then, the cells were washed with PBS, and fresh medium containing 10% FBS was added. After incubation for 24 hours at 37 °C, the cells were fixed and photographed. (**F**) The transwell migration assay showed the different migratory responses of the two stable cell lines. The cell lines were added to the upper chamber and 500 μl of appropriate medium supplemented with 10% FBS was added to the lower chamber. After incubation for 24 hours at 37 °C, the chambers were stained and photographed. Three random fields were analyzed for each chamber.

The IAV NS1 protein is a multifunctional protein that has been previously shown to interact with many host cell factors and play important roles in virus replication^54^. To better understand the roles of NS1 in influenza virus pathogenesis, we generated stable cell lines (P1, P2 and P3) that over-expressed the NS1 protein. The N1, N2 and N3 cell lines were used as the negative controls. NS1 expression was confirmed using western blotting and immunofluorescence assays (Fig. 1A,B). Then, we observed the phenotypic changes in these two stable cell lines (Fig. 1C). The spreading lamellipodia were visible in the control N1, N2 and N3 cell lines, but were retracted in the P1, P2 and P3 cell lines. Figure 1D shows the different percentages of the cell spreading area. Thus, NS1 inhibited the formation of cellullar lamellipodia.

Because the lamellipodia is very important for the cell migration ability^55^, we performed wound healing and transwell assays to investigate whether NS1 over-expression inhibites cell migration. Wounds were made in a confluent cell layer with a sterile pipette tip, as shown in Fig. 1E. The control cell lines exhibited better healing abilities than the NS1 over-expression cell lines. Further, the transwell migration assay was performed to determine the differences between the two cell lines. As shown in Fig. 1F, there was a significant reduction in the migration of the P1, P2 and P3 cells from the small holes compared to the control N1, N2 and N3 cells, which indicated that over-expression of NS1 significantly inhibited the cell migration speed. Taken together, our results indicated that the NS1 protein inhibited lamellipodia formation and cell motility.

### NS1 down-regulates Rac1 activity

**Figure 2:**
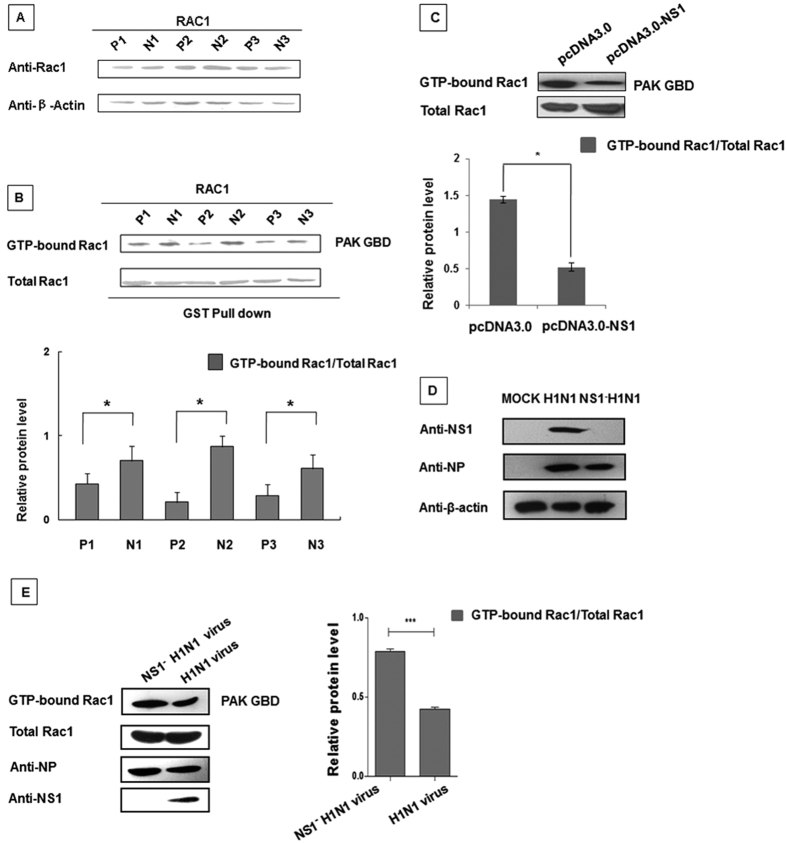
NS1 down-regulates Rac1 activity. (**A**) There was no significant difference in the Rac1 protein expression level between the two different cell lines. The two cell lines were seeded into six-well plates. After 24 hours, the Rac1 protein expression levels were assessed in the whole cell lysates using the indicated antibodies as appropriate. (**B**) GTP-bound Rac1 levels were lower in the NS1 over-expressing cell lines than in the negative control cell lines. Lysates from the two stable cell lines were incubated with equal amounts of GST-PAK bound glutathione Sepharose 4B beads. After washing, the bound proteins were analyzed by western blotting using an anti-Rac1 antibody. The protein band intensity was measured using the ImageJ software (NIH). Data represent the mean fold change ± S.D. of three independent experiments (*p < 0.05 relative to the control group). (**C**) Rac1 activity was reduced in 293T cells transfected with pcDNA3.0-flag-NS1. 293T cells were co-transfected with Myc-tagged Rac1 and the indicated plasmids. After 30 hours, the whole cell lysates were subjected to the GST-PAK pull down assay, followed by western blotting analysis using an anti-Myc antibody. The protein band intensity was measured using the ImageJ software (NIH). Data represent the mean fold change ± S.D. of three independent experiments (*p < 0.05 relative to the control group). (**D**) Confirmation of the recombinant virus (NS1^−^ H1N1). A549 cells were infected with the H1N1 virus or the recombinant virus lacking the NS1 gene (NS1^−^ H1N1) at an MOI of 0.5 or left uninfected. After 24 hours, whole cell lysates were subjected to western blotting. (**E**) Rac1 activity in wild type virus-infected A549 cell was lower than the activity in cells infected with the mutated virus. A549 cells were infected with the A/WSN/33 H1N1 virus and the mutated NS1^−^ H1N1 virus (MOI = 0.5) for 24 hours. Then, the cell lysates were collected and subjected to the GST-PAK pull down assay followed by western blotting analysis using an anti-Rac1 antibody. The protein band intensity was measured using the ImageJ software (NIH). Data represent the mean fold change ± S.D. of three independent experiments (***p < 0.001 relative to the control group).

Our previous results showed that NS1 inhibited cellular lamellipodia formation and motility. Because the Rac1 protein is essential for platelet lamellipodia formation^30^, we hypothesized that NS1 might regulate the Rac1 protein functions. To test this hypothesis, we used the western blotting and GST-pull down techniques to analyze Rac1 expression and activity in the NS1 over-expressing cell lines. As shown in Fig. 2A, the Rac1 expression levels were unchanged by NS1 over-expression. Previous studies demonstrated that Rac1’s function as a molecular switch was regulated by the GTP/GDP exchange^56^. Consequently, we measured the Rac1-GTP level. The Rac1-GTP level was significantly reduced in the NS1 over-expressing P1, P2 and P3 cell lines compared to the control N1, N2 and N3 cell lines (Fig. 2B). To validate this observation, the NS1 protein was transfected into 293T cells, and endogenous Rac1 activity was assessed. As shown in Fig. 2C, transient over-expression of NS1 also resulted in a significant reduction in the GTP-bound Rac1 level. Therefore, we generated an influenza virus A/WSN/33 strain that lacked the NS1 gene (NS1^−^ H1N1 virus) (Fig. 2D) and infected A549 cells with the wild type (wt) H1N1 or NS1^−^ H1N1 virus. As shown in Fig. 2E, the Rac1 activity in the wt virus-infected cells was significantly lower than the activity in the cells infected with the mutated virus. Therefore, the NS1 protein suppressed Rac1 activity and reduced the Rac1-GTP level, although the Rac1 protein expression level remained unaffected.

### NS1 protein directly interacts with cellular Rac1

**Figure 3:**
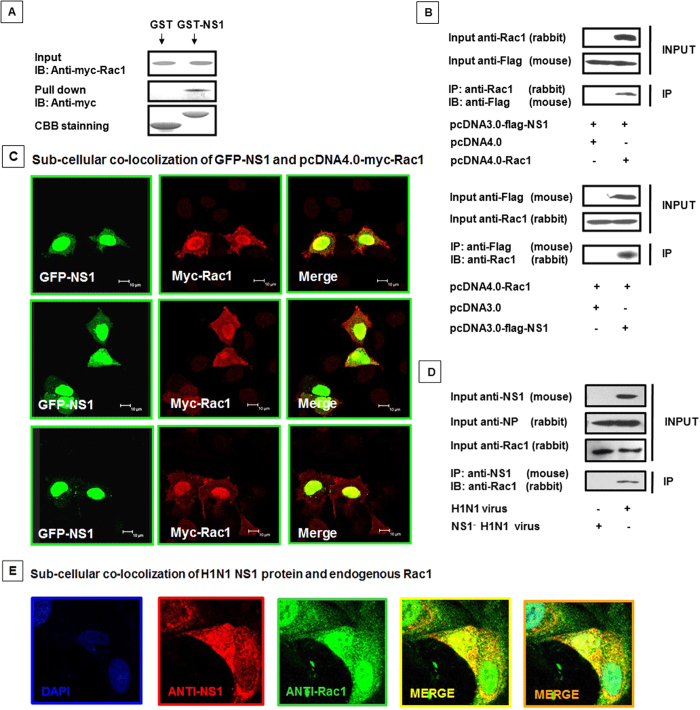
NS1 interacts with Rac1 *in vivo*. (**A**) GST-pull down assay showing the interaction between NS1 and Rac1. Whole 293T cell lysates transfected with Myc-tagged Rac1 were incubated with an equal amount of GST or GST-NS1 bound to glutathione-Sepharose 4B beads, followed by IB using the anti-Myc antibody. CBB, Coomassie brilliant blue staining. (**B**) Co-immunoprecipitation of Flag-NS1 and Myc-Rac1 in 293T cells. The 293T cells were co-transfected with Flag-tagged NS1 and pcDNA4.0 or pcDNA4.0-myc-Rac1; Myc-tagged Rac1 and pcDNA3.0 or pcDNA3.0-flag-NS1. Rac1 antibodies (rabbit) were applied to the cell lysates for IP, followed by IB with Flag (mouse); alternatively, the IP was performed with a Flag antibody (mouse) followed by IB with Rac1 (rabbit). (**C**) Co-localization of GFP-NS1 and Myc-Rac1 in A549 cells. A549 cells were co-transfected with pEGFP-NS1 and pcDNA4.0-myc-Rac1 for 28 hours and then fixed, permeabilized, and stained for Myc-Rac1 (red). Yellow indicates overlap. (**D**) Co-immunoprecipitation of virus NS1 and endogenous Rac1 in A549 cells. A549 cells were infected with the A/WSN/33 H1N1 virus and the mutated NS1^−^ H1N1 virus (MOI = 0.5) for 24 hours, and the cell lysates were immunoprecipitated and immunoblotted with the indicated antibodies. (**E**) Co-localization of viral NS1 and endogenous Rac1 in A549 cells. A549 cells were infected with the A/WSN/33 H1N1 virus (MOI = 0.1) for 24 hours and then fixed, permeabilized, and stained with DAPI (blue), anti-NS1 antibody (red), and anti-Rac1 antibody (green). Yellow indicates overlap with red and green. Orange indicates overlap with blue, red and green.

Our previous results demonstrated that NS1 over-expression and wt virus infection resulted in a reduced level of GTP-bound Rac1, next we investigated whether NS1 regulates Rac1 activity through a direct interaction. As shown in Fig. 3A, the use of GST fusion constructs of the NS1 protein in the pull-down assays demonstrated that the NS1 protein interacted with Rac1. We further confirmed the direct interaction of NS1 and cellular Rac1 via co-immunoprecipitation in transfected cells (Fig. 3B). Co-transfection with pEGFP-NS1 and pcDNA4.0-myc-Rac1 resulted in a complete overlay of the GFP and red anti-myc-Rac1 immunofluorescence, indicating the co-localization of NS1 and Rac1 (Fig. 3C). Moreover, we infected A549 cells with the wt H1N1 and NS1^−^ H1N1 viruses, immunoprecipitation of Rac1 only detected in wt virus but not NS1^−^ H1N1 virus infection (Fig. 3D), which indicated that the NS1 protein directly interacted with the cellular Rac1 protein. The co-localization of viral NS1 and endogenous Rac1 in the wt virus-infected A549 cells was also visualized by confocal microscopy (Fig. 3E). Collectively, our results indicated that the NS1 protein directly interacted with Rac1 in the cytoplasm and nucleus.

### NS1 suppresses Rac1 activity by inhibiting its ubiquitination

**Figure 4:**
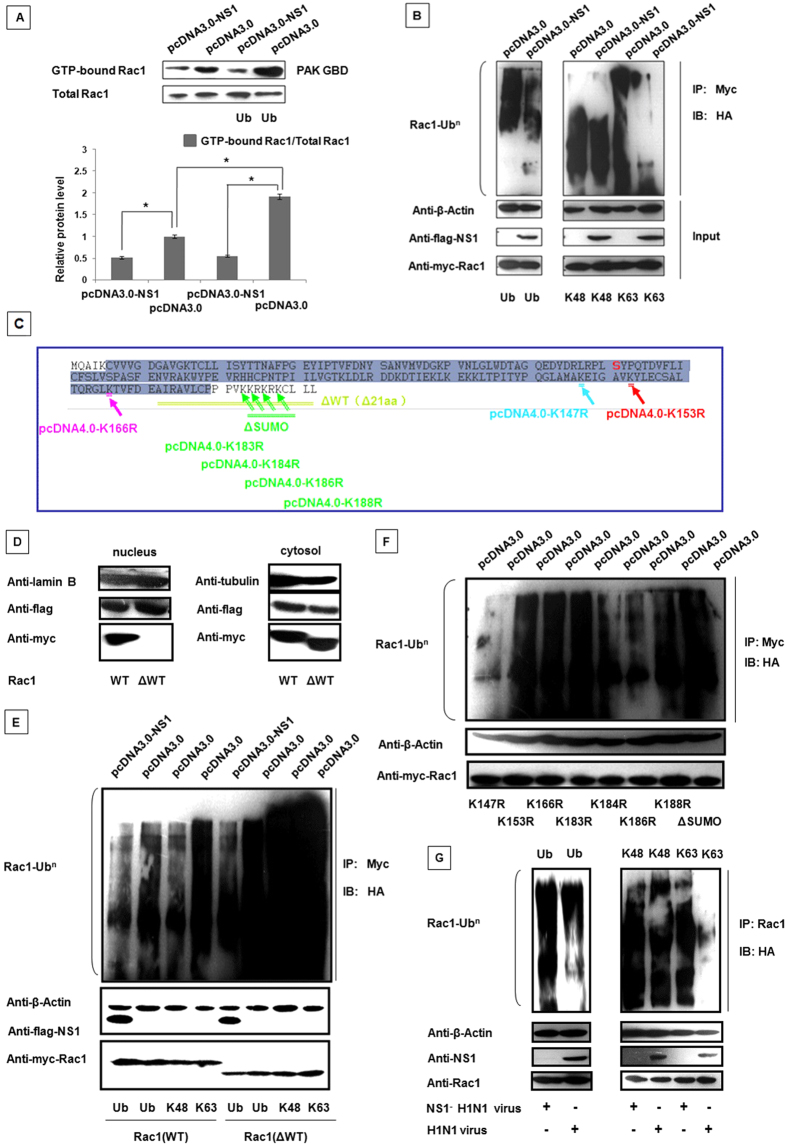
NS1 down-regulates Rac1 activity by inhibiting ubiquitination. For the ubiquitination analysis, the proteasome inhibitor MG-132 was added to the cells for 6 h after transfection for 24 hours. (**A**) The amount of GTP-bound Rac1 was increased by the ubiquitin modification, and reduced by the NS1 protein expression. 293T cells were co-transfected with Myc-tagged Rac1, HA-Ub and pcDNA3.0 or pcDNA3.0-flag-NS1 as indicated. After 30 hours, whole cell lysates were subjected to the GST-PAK pull down assay followed by western blotting using the anti-Myc antibody. (**B**) The NS1 protein inhibited Rac1 activity through interference with its K63-linked poly-ubiquitination. 293T cells were co-transfected the plasmids as indicated. An anti-Myc antibody was used to precipitate Rac1, and western blotting was using an HA-specific antibody. (**C**) The amino acid sequence of Rac1 and its mutation sites. The Rac1 mutations ΔSUMO contained mutations in the K183, K184, K186 and K188 residues. (**D**) Validation of the finding that pcDNA4.0-myc-Rac1 ΔWT primarily expressed in the cytoplasm. 293T cells were co-transfected with pcDNA3.0-flag-NS1 and pcDNA4.0-myc-Rac1 WT or pcDNA4.0- myc-Rac1 ΔWT. Nuclear and cytoplasmic proteins were extracted and assayed by western blotting with the indicated antibodies. Lamin B1 and tubulin were used as loading controls for the nuclear and cytoplasmic fractions, respectively. (**E**) Deletion of the Rac1 C-terminal NLS sequence enhanced its ubiquitination. 293T cells were transfected with the plasmids as indicated. An anti-Myc antibody was used to precipitate the cell lysates, and a western blotting was using an anti-HA antibody. (**F**) The ubiquitin sites of the Rac1 protein. 293T cells were co-transfected with HA-Ub, pcDNA3.0 and mutated Rac1 plasmids. An anti-Myc antibody was used to precipitate the lysates, and western blotting was using an anti-HA antibody. (**G**) The wild type virus inhibited Rac1 activity through interference with its K63-linked poly-ubiquitination. 293T cells were transfected with HA-Ub, HA-K48R and HA-K63R as indicated for 24 hours. Then, the cells were infected with the wt virus and the mutated virus (MOI = 0.5) for another 24 hours. An anti-Rac1 antibody was used to precipitate endogenous Rac1 from the cell lysates, and western blotting was using an HA-specific antibody.

Recent studies suggested that Rac1 was modulated by ubiquitination and SUMOylation. The ubiquitin pathway is the most common method used by cells to regulate protein activation, localization and function^57^,^58^. Therefore, to identify whether ubiquitination was involved in the NS1-mediated regulation of Rac1, we co-transfected Myc-Rac1 and HA-Ub with pcDNA3.0-NS1 or pcDNA3.0 in 293T cells. Then, a GST-pull down assay was performed on these samples. As shown in Fig. 4A, the presence of NS1 decreased Rac1 activity. Moreover, Rac1 ubiqitination enhanced the Rac1-GTP level, whereas NS1 suppressed this process. Thus, our data indicated that Rac1 activity could be modulated by ubiquitination.

Different linkage-specific ubiquitin chain modifications have been shown to mediate distinct cellular events. K63-linked polyubiquitin chains are involved in the activation of antiviral signaling pathways and the regulation of a variety of nonproteolytic cellular functions, including gene transcription, subcellular localization, protein activity, intracellular trafficking and viral budding^59^,^60^. Conversely, K48-linked polyubiquitin chains generally target proteins for proteasomal degradation^61^,^62^. Because our results indicated that NS1 inhibited Rac1 ubiquitination, next we investigated the specific types of polyubiquitin chain linkages modulated by NS1. As shown in Fig. 4B, Rac1 ubiquitination was reduced by the co-transfection of K63-linked polyubiquitin chains, whereas no difference was noted with the co-transfection of K48-linked polyubiquitin chains, indicating that Rac1 was associated with K63-linked ubiquitination.

The C-terminus of Rac1 is crucial for binding to several regulatory proteins and the proper localization of Rac1 in the plasma membrane, and the C-terminal 21 aa of Rac1 were confirmed as the nuclear localization sequence (NLS)^58^. To confirm the location of the ubiquitin reaction in the cell, we constructed a mutant plasmid with a deletion of the C-terminal 21aa of Rac1 (called Rac1 ΔWT). The mutation plasmid is shown in Fig. 4C (Rac1 amino acid sequence) colored in yellow-green. Figure 4D showed that the Rac1 ΔWT protein was only expressed in the cytoplasm, which was consistent with previous reports^63^,^64^. Then, we used the wt Rac1 protein as the control to detect the degree of ubiquitination the Rac1 ΔWT protein. As shown in Fig. 4E, the degree of ubiquitination was increased by the deletion of the C-terminal 21 aa, which indicated that the ubiquitination reactions primarily occurred in the cytoplasm. To accurately determine the Rac1 ubiquitination sites, we predicted possible ubiquitination sites (BDM-PUB, K147, K153, K166 and K183/184/186/188) and mutated the lysine to arginine at those sites (Fig. 4C). When lysine 147 was mutated to arginine (K147R), the ubiquitination nearly disappeared, which indicated that K147 was the major site in Rac1 for ubiquitin modification, although the K184 and K186 sites also underwent ubiquitination to a lesser extent (Fig. 4F). Then, 293T cells were transfected with HA-Ub, HA-K48R and HA-K63R for 24 hours and infected with the wt H1N1 and NS1^−^ H1N1 viruses. The anti-Rac1 antibody was used to precipitate endogenous Rac1 from the cell lysates, and western blotting of the precipitates was conducted using an HA-specific antibody. We found that the wt virus inhibited Rac1 activity through interference with its K63-linked poly-ubiquitination compared to the NS1^−^ H1N1 virus (Fig. 4G). These data indicated that NS1 suppressed Rac1 activity by inhibiting K63-linked ubiquitination.

### NS1 inhibits Rac1 SUMOylation, resulting in reduced Rac1-GTP levels

**Figure 5:**
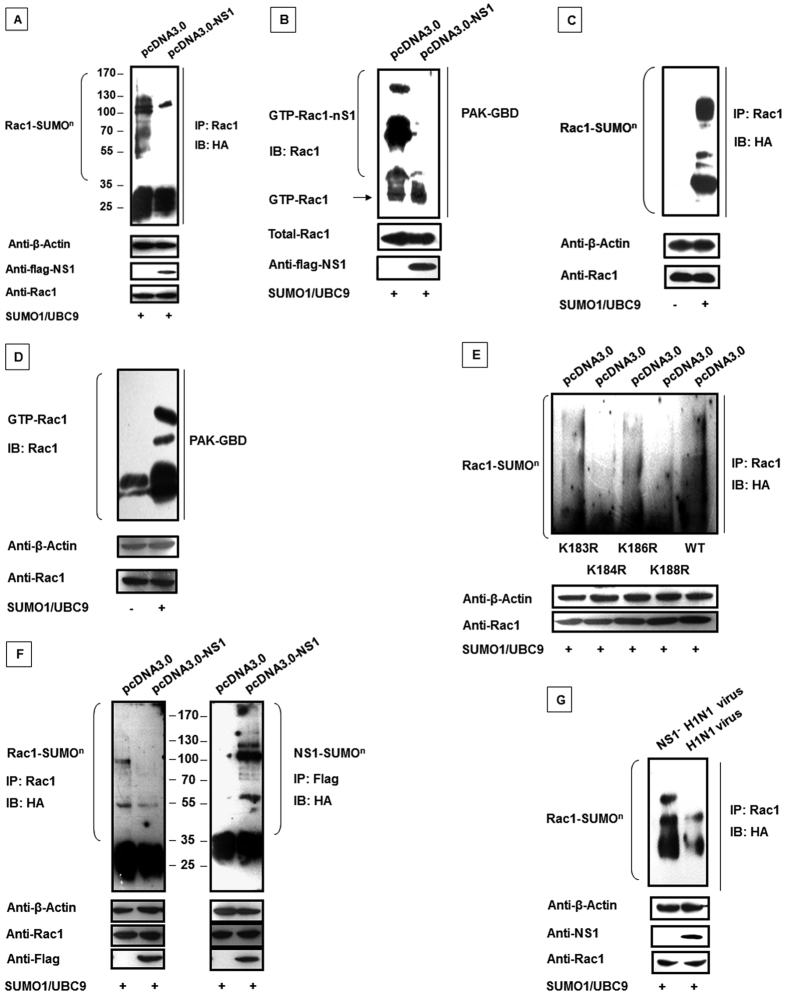
NS1-mediated inhibition of Rac1 SUMOylation results in reduced Rac1 activity. (**A**) NS1 inhibited Rac1 SUMOylation. 293T cells were co-transfected with pcDNA3.0 or pcDNA3.0-flag-NS1 with pcDNA4.0-myc-Rac1, pXJ40-HA-SUMO-1 and UBC-9 as indicated. Anti-Rac1 antibodies were used to precipitate the cell lysates, and western blotting was conducted using HA-specific antibodies. (**B**) NS1 decreases SUMOylated GTP-bound Rac1. 293T cells were co-transfected with pcDNA3.0 or pcDNA3.0- flag-NS1 with pcDNA4.0- myc-Rac1, pXJ40-HA-SUMO-1 and UBC-9 as indicated. After 30 hours, the whole cell lysates were subjected to the GST-PAK pull down assay followed by western blotting using a Rac1-specific antibody. (**C**) Rac1 SUMOylation. 293T cells were co-transfected with pcDNA4.0-myc-Rac1, pXJ40-HA-SUMO-1 and UBC-9 as indicated. Anti-Rac1 antibodies were used to precipitate the cell lysates, and western blotting was conducted using HA-specific antibodies. (**D**) Rac1 SUMOylation can increase Rac1 activity. 293T cells were co-transfected with pcDNA4.0- myc-Rac1, pXJ40-HA-SUMO-1 and UBC-9 as indicated. After 30 hours, the whole cell lysates were subjected to the GST-PAK pull down assay followed by western blotting using a Rac1-specific antibody. (**E**) The SUMOylation sites in the Rac1 protein. 293T cells were co-transfected with pcDNA3.0, pXJ40-HA-SUMO-1, UBC-9, and the mutated Rac1 plasmids as indicated. Rac1 antibodies were used for precipitation of the lysates, and western blotting was performed with HA-specific antibodies. (**F**) Competition for SUMOylation by the NS1 protein inhibited Rac1 SUMOylation. 293T cells were co-transfected with pcDNA3.0 or pcDNA3.0-flag-NS1 with pcDNA4.0- myc-Rac1, pXJ40-HA-SUMO-1 and UBC-9 as indicated. Anti-Rac1 antibodies were used to precipitate the cell lysates, and western blotting was performed using HA-specific antibodies. (**G**) The wild type virus can inhibit Rac1 SUMOylation. 293T cells were co-transfected with pcDNA3.0 or pcDNA3.0- flag-NS1 with pcDNA4.0- myc-Rac1, pXJ40-HA-SUMO-1 and UBC-9 as indicated. Then, the cells were infected with the A/WSN/33 H1N1 virus and the mutated NS1^−^ H1N1 virus (MOI = 0.5) for another 24 hours. Anti-Rac1 antibodies were used to precipitate the cell lysates, and western blotting was conducted using HA-specific antibodies.

Castillo-Lluva *et al*. recently demonstrated that GTP-bound Rac1 levels could be regulated by SUMOylation, thereby stimulating cell lamellipodia, cell migration and invasion^25^. We confirmed these results in our experiments. We found that Rac1 SUMOylation was reduced following NS1 over-expression in transfected 293T cells (Fig. 5A). And with the presence of SUMO and UBC9, we observed that the NS1 protein inhibited SUMOylated GTP-bound Rac1 (Fig. 5B). Additionally, we detected the effect of SUMOylation on Rac1 activity. Figure 5C,D showed that GTP-bound Rac1 was increased by the presence of SUMO and UBC9. Next, we investigated potential SUMOylation sites in Rac1. According to the study by Castillo-Lluva and colleagues, SUMO-1 was conjugated to lysine residues 183, 184, 186 or 188 in nearly 95% of SUMOylated Rac1; therefore, we selected these amino acid residues for analysis as potential Rac1 SUMOylation sites. As shown in Fig. 5E, the K184 and K188 sites were the key residues responsible for the SUMO modifications. Furthermore, we assessed the role of NS1 on Rac1 SUMOylation. Figure 5F shows the results obtained after the precipitation of flag-tagged NS1 and probing with the HA antibody to detect the precipitated NS1. We found that NS1 itself could be SUMOylated, which confirmed a previous report^65^. Then, the Rac1 SUMOylation assay was performed following infection with the wt H1N1 and NS1^−^ H1N1 viruses. The Rac1 SUMOylation level in the wt virus-infected cells were lower than the level in the cells infected with the mutated virus (Fig. 5G). The results were consistent with NS1 protein over-expression. Cumulatively, these results strongly indicated that competitive inhibition of Rac1 SUMOylation by NS1 decreased the Rac1-GTP level.

### Suppression of Rac1 activity results in arrested cell migration and altered cell morphology

**Figure 6:**
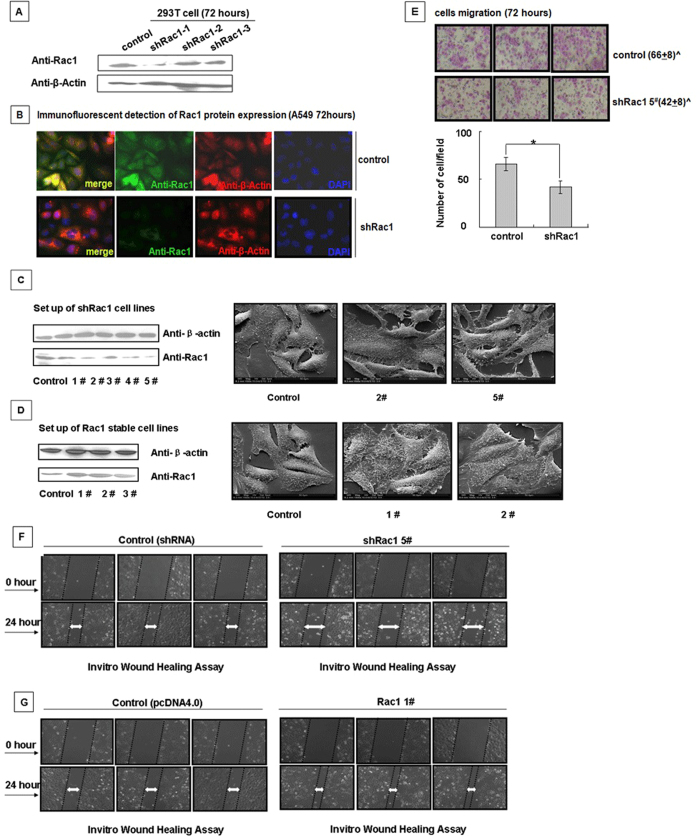
Generation of the shRac1 cell line and scanning electron microscopy to monitor cell morphology and migration changes. (**A**) Selection of shRNA for Rac1. 293T cells were transfected with the indicated shRNA (sequences in Table 1) for 72 hours, and Western blotting was used to select effective shRac1 plasmids. (**B**) The efficacy of Rac1-specific shRac1-1 was confirmed by the immunofluorescence assay. A549 cells transfected with control shRNA and shRac1-1 for 28 hours were fixed, permeabilized, and stained for Rac1 (green), β-actin (red) and DAPI (blue). (**C,D**) Generation of shRac1 and Rac1 stable cell lines. A549 cells were transfected with shRac1-1 and pcDNA4.0-myc-Rac1 and then selected with 800 mg/L of G418. A western blotting analysis verified the stably transfected cell strains, and the SEM pictures showed differences in the morphology and microstructures of these two different stable cell lines. (**E**) Interference with Rac1 resulted in impaired cell migration. Transwell migration assay demonstrated the migratory response of the Rac1-specific shRNA cell line. (**F,G**) Comparison of the migration ability between the Rac1 and shRac1 cell lines. A wound healing assay was performed to determine the migration ability of the shRac1 cell line and the Rac1 over-expressing cell line.

To determine whether Rac1 is the key regulator that mediates NS1-induced cell morphological alterations, we designed shRNA sequences specific for Rac1. Based on the depletion efficiency of the Rac1-specific shRNAs (Fig. 6A,B), we chose shRac1-1 as the Rac1 interfering sequence for the following experiments. Stable A549 cell lines expressing shRac1-1 and Rac1 were generated and confirmed by western blotting analysis (Fig. 6C,D). The 2^#^ and 5^#^ shRac1 and the 1^#^ and 2^#^ Rac1 over-expression cell lines were chosen and photographed by SEM. They showed that interference with Rac1 led to the contraction of the cell lamellipodia, whereas over-expression of Rac1 promotes lamellipodial spreading, which is concordant with the cell morphology of the stable NS1 cell lines (Fig. 1C). As shown in Fig. 6E (the upper part of Fig. 6), the numbers of migrated cells from the shRac1 cell lines were lower than the numbers of migrated cells from the control cell lines, similar to the results obtained for the NS1 over-expressing cell lines P1, P2 and P3 shown in Fig. 1F. The wound healing assay indicated that interference with Rac1 protein expression resulted in the inhibition of cell migration, whereas over-expression of Rac1 promoted cell migration and sped cellular scratch healing (Fig. 6F,G). Thus, the above data indicated that the NS1-induced retraction of lamellipodia and inhibition of cell migration might be due to direct suppression of the normal function of Rac1.

### The impact of Rac1 on H1N1 viral replication

**Figure 7:**
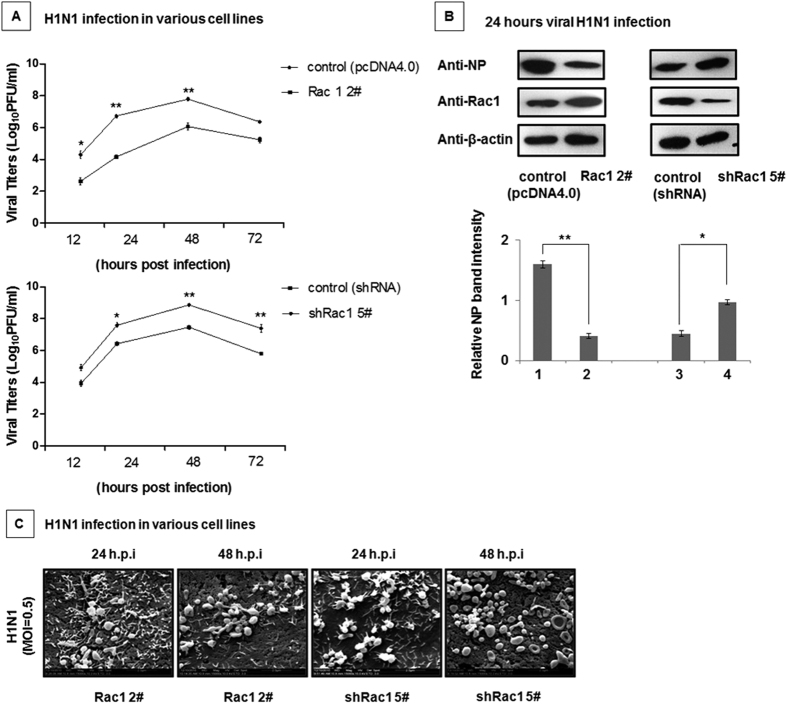
The impact of Rac1 on H1N1 viral replication. (**A**) Rac1 suppressed H1N1 viral replication. The A549-Rac1 2^#^ and A549-shRac1 5^#^ cell lines and the control cell lines were infected with the H1N1 virus at an MOI of 0.5. The cell culture supernatants were collected at the indicated times post infection, and the plaque assay was used to measure the virus titers. Data represent the mean fold change ± S.D. of three independent experiments (*p < 0.05 relative to the control group). (**B**) Over-expression of Rac1 decreased the NP protein content. The A549-Rac1 2^#^ and A549-shRac1 5^#^ cell lines and the control cell lines were infected with the H1N1 virus as indicated at an MOI of 0.5. After 24 hours, the cells were collected and analyzed by western blotting with the indicated antibodies. Relative NP band intensities (relative to β-actin) were measured using the ImageJ software (NIH). Data represent the mean fold change ± S.D. of three independent experiments (*p < 0.05 relative to the control group). (**C**) Rac1 protein over-expression inhibited virus replication, whereas interference with Rac1 promoted viral replication. The A549-Rac1 2^#^ and A549-shRac1 5^#^ cell lines were infected with the H1N1 virus for 24 and 48 hours. Both stable cell lines were examined by SEM after infection.

Rac1 was reported to suppress A/PR/8/34 virus (PR8) replication^35^. Due to the different characteristics between the A/PR/8/34 and A/WSN/33 influenza viruses, we investigated whether Rac1 also inhibited A/WSN/33 viral replication. To answer this question, the A549-Rac1 2^#^ cell line, A549-shRac1 5^#^ cell line, and the control were infected with the WSN virus at an MOI of 0.5. The virus titers were determined via plaque assay. As shown in Fig. 7A, Rac1 over-expression inhibited viral replication, whereas interfering with Rac1 facilitated viral replication. The impact of Rac1 on H1N1 replication was confirmed in different stable cell lines by analyzing the viral NP protein level (Fig. 7B). Next, we observed the replication of the wt H1N1 virus in both the Rac1 over-expressing and the Rac1 interfered cell lines by SEM (Fig. 7C). The viral particles in the Rac1 over-expressing cell lines was lower than that in the Rac1 interfering cell lines, which was the same as the result obtained in the plaque assay (Fig. 7A). Thus, Rac1 can inhibit H1N1 virus A/WSN/33 replication.

### The impact of Rac1 on NS1^−^ H1N1 viral replication

**Figure 8:**
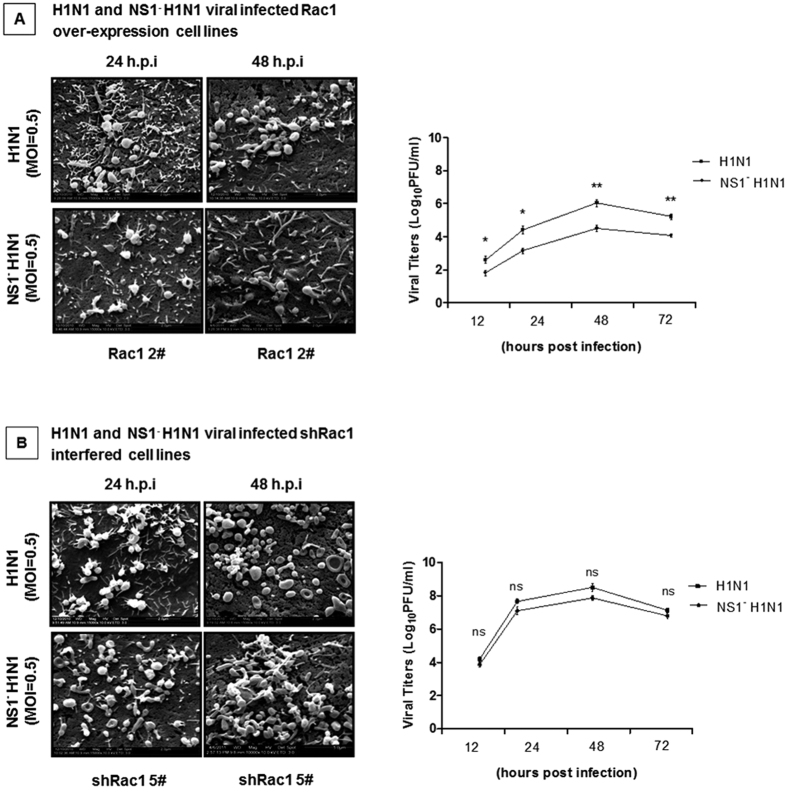
The impact of Rac1 on NS1^−^ H1N1 viral replication. (**A**) The H1N1 virus particles were significantly higher than the NS1^−^ H1N1 virus particles in the Rac1 over-expressing cell lines. The A549-Rac1 2^#^ cell line was infected with the H1N1 virus and the NS1^−^ H1N1 mutant virus at an MOI of 0.5. SEM was performed on the cell lines after they were infected for 24 and 48 hours. The cell culture supernatants were collected at the indicated times post infection, and the plaque assay was used to measure the virus titers. Data represent the mean fold change ± S.D. of three independent experiments (*p < 0.05, **p < 0.005 relative to the control group). (**B**) The NS1^−^ H1N1 virus particles was approximately the same as the wild type virus particles in the Rac1-interfered cell lines. The A549-shRac1 5^#^ cell line was infected with the H1N1 virus and NS1^−^ H1N1 mutant virus at an MOI of 0.5. SEM was performed on the cell lines after infection for 24 and 48 hours. The cell culture supernatants were collected at the indicated times post infection, and the plaque assay was used to measure the virus titers. Data represent the mean fold change ± S.D. of three independent experiments.

Our previous results indicated that the IAV viral NS1 protein could inhibit the normal function of the Rac1 protein, whereas interference with Rac1 resulted in increased viral replication. Next, SEM and plaque assays were performed following infection with the wt H1N1 and NS1^−^ H1N1 viruses. Figure 8A shows that the H1N1 viral particles in the Rac1 over-expressing cell lines was greater than the NS1^−^ H1N1 virus particles. This result was validated by the plaque assay. This phenomenon suggested that the NS1 protein plays an important role in enhancing viral replication by inhibiting Rac1 activity. We further tested the Rac1 interfering cell lines infected with the wild type and mutant viruses. As shown in Fig. 8B, there was no significant difference in the amount of virus particles between the wt and mutant viruses in the absence of Rac1, which indicated that the NS1 protein augmented virus replication partially by inhibiting Rac1 activity. Overall, the A/WSN/33 virus H1N1 inhibits Rac1 activity to facilitate viral replication via the viral NS1 protein.

## Discussion

IAVs rely on host factors to assist their propagation within the host. The identification of these cellular proteins will help to elucidate the mechanisms by which the viruses acquire the potential to become pandemic in the human population. In our study, we generated NS1 over-expressing cell lines, we found that NS1 over-expression induced pseudopodial retraction and inhibited cell migration. Previous studies reported that the small GTPase Rac1 was involved in cytoskeletal rearrangements and lamellipodia formation and played key roles during virus entry, replication and release^33^,^66^,^67^, thus, Rac1 inhibition leads to enhanced virus production^35^. Therefore, we measured the Rac1 protein expression levels in our NS1 over-expressing cell lines. The Rac1 expression level remained unchanged, but its activity was significantly reduced with NS1 protein over-expression. We demonstrated that Rac1 ubiquitination and SUMOylation were inhibited by NS1 and that the inhibition of these post-translational modifications led to decreased Rac1 activity and enhanced viral replication.

In addition to modulation by GEFs, GAPs and GDIs, Rac1 is subject to modifications^68^. Some studies have reported that the regulation of Rac1 drives diverse cellular processes during virus replication^69^,^70^, and the ubiquitination and SUMOylation of Rac1 have been suggested to be involved in pseudopodial growth and the inhibition of virus replication^71^,^72^. Currently, little is known regarding the molecular mechanisms regulated by Rac1. Here, our studies revealed a previously unknown function of the viral NS1, which directly interacted with the Rac1 protein. Our experimental results showed that the Rac1 protein can be modified by Lys63-linked ubiquitination and SUMO1-conjugated SUMOylation. Lysine 147 of Rac1 was most likely the main acceptor site for ubiquitin, which was previously reported by Orane Visvikis^58^; however, we demonstrated that this site of the Rac1 protein was primarily responsible for K63-linked ubiquitination during influenza virus infection. In addition to this finding, the lysine 184 and 186 sites also contributed to ubiquitination at a certain extent. Lysine 184 and 188 are the sites for Rac1 SUMOylation, which is in accordance with the data presented by Castillo-Lluva^25^. Importantly, Rac1 can inhibit viral replication, as demonstrated with the A/PR/8/34 virus by Ehrhardt *et al*.^35^. Our results clearly showed that the H1N1 A/WSN/33 virus inhibited Rac1 activity to facilitate viral replication via the direct interaction of the viral NS1 protein and the cellular Rac1 protein.

Influenza A virus takes advantage of host posttranslational modifications for its own benefit. The nonstructural NS1 protein is the first discovered and the most frequent SUMO target of IAV, which is SUMOylated to facilitate viral replication^73^. Our results showed that the SUMOylation of the small GTP-binding protein Rac1 was competitively inhibited by the viral NS1 protein in the host cell, indicating that IAV profoundly relied on SUMOylation to survive in host cells.

Consistent with our previous observations, the ability of NS1 to regulate viral replication by inhibiting Rac1 activity provides new insights into viral replication. This finding demonstrates the importance of the NS1 protein in regulating the host cellular response and paves the way toward new potential therapeutic targets.

## Additional Information

**How to cite this article**: Jiang, W. *et al*. Suppression of Rac1 Signaling by Influenza A Virus NS1 Facilitates Viral Replication. *Sci. Rep.* **6**, 35041; doi: 10.1038/srep35041 (2016).

**Publisher’s note**: Springer Nature remains neutral with regard to jurisdictional claims in published maps and institutional affiliations.

## Figures and Tables

**Table 1:** shRNA primer sequences.

Name	Sequences (5′-3′)
shRNA(control)	Forward: ACCGCATTCCGGAATTCCGGCACCTTCCTGTCA GTGCCGGAATTCCGGAATGCTTTTTC
shRNA(control)	Reverse: TGCAGAAAAAGCATTCCGGAATTCCGGCACTGACAGGAAGGTGCCGGAATTCCGGAATG
shRac1-1	Forward: ACCGGGTCTGTGAGGTTCTGTACTTCCTGTCATACAGAACCTCACAGACCCTTTTTC
shRac1-1	Reverse: TGCAGAAAAAGGGTCTGTGAGGTTCTGTA TGACAGGAAGTACAGAACCTCACAGACC
shRac1-2	Forward: ACCGTGTTCCCGACATAACATTCTTCCTGTCAAATGTTATGTCGGGAACAC TTTTTC
shRac1-2	Reverse: TGCAGAAAAAGTGTTCCCGACATAACATTTGACAGGAAGAATGTTATGTCGGGAACA
shRac1-3	Forward: ACCGGTTACACAACCAATGCATCTTCCTGTCAATGCATTGGTTGTGTAACTTTTTC
shRac1-3	Reverse: TGCAGAAAAAGTTACACAACCAATGCATTGACAGGAAGATGCATTGGTTGTGTAAC
